# Chemical Probes to Control and Visualize Lipid Metabolism in the Brain

**DOI:** 10.1021/acs.accounts.2c00521

**Published:** 2022-10-25

**Authors:** Jeroen M. Punt, Daan van der Vliet, Mario van der Stelt

**Affiliations:** Department of Molecular Physiology, Leiden Institute of Chemistry, Leiden University & Oncode Institute, Einsteinweg 55, Leiden 2333 CC, The Netherlands

## Abstract

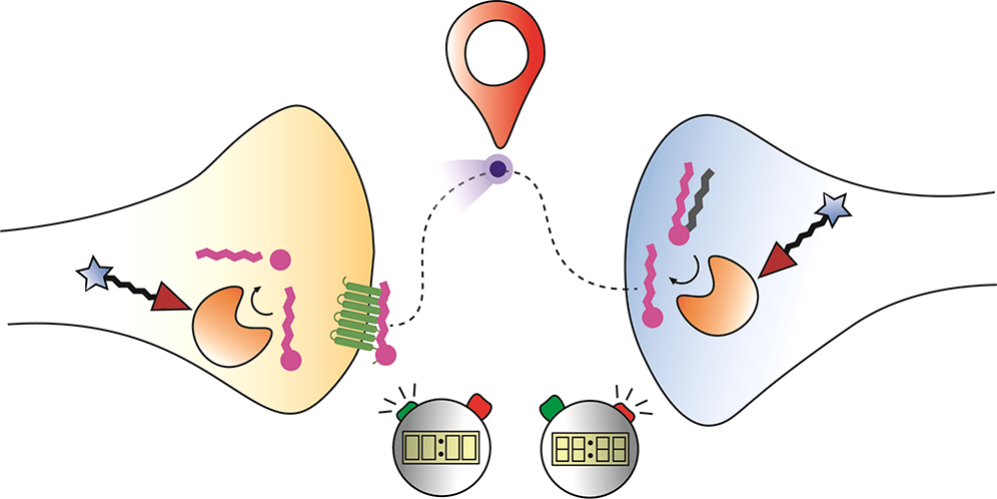

Signaling lipids, such as the endocannabinoids, play an important role in the brain. They regulate synaptic transmission and control various neurophysiological processes, including pain sensation, appetite, memory formation, stress, and anxiety. Unlike classical neurotransmitters, lipid messengers are produced on demand and degraded by metabolic enzymes to control their lifespan and signaling actions. Chemical biology approaches have become one of the main driving forces to study and unravel the physiological role of lipid messengers in the brain. Here, we review how the development and use of chemical probes has allowed one to study endocannabinoid signaling by (i) inhibiting the biosynthetic and metabolic enzymes; (ii) visualizing the activity of these enzymes; and (iii) controlling the release and transport of the endocannabinoids. Activity-based probes were instrumental to guide the discovery of highly selective and in vivo active inhibitors of the biosynthetic (DAGL, NAPE-PLD) and metabolic (MAGL, FAAH) enzymes of endocannabinoids. These inhibitors allowed one to study the role of these enzymes in animal models of disease. For instance, the DAGL–MAGL axis was shown to control neuroinflammation and the NAPE-PLD–FAAH axis to regulate emotional behavior. Activity-based protein profiling and chemical proteomics were essential to guide the drug discovery and development of compounds targeting MAGL and FAAH, such as ABX-1431 (Lu AG06466) and PF-04457845, respectively. These experimental drugs are now in clinical trials for multiple indications, including multiple sclerosis and post-traumatic stress disorders. Activity-based probes have also been used to visualize the activity of these lipid metabolizing enzymes with high spatial resolution in brain slices, thereby showing the cell type-specific activity of these lipid metabolizing enzymes. The transport, release, and uptake of signaling lipids themselves cannot, however, be captured by activity-based probes in a spatiotemporal controlled manner. Therefore, bio-orthogonal lipids equipped with photoreactive, photoswitchable groups or photocages have been developed. These chemical probes were employed to investigate the protein interaction partners of the endocannabinoids, such as putative membrane transporters, as well as to study the functional cellular responses within milliseconds upon irradiation. Finally, genetically encoded sensors have recently been developed to monitor the real-time release of endocannabinoids with high spatiotemporal resolution in cultured neurons, acute brain slices, and in vivo mouse models. It is anticipated that the combination of chemical probes, highly selective inhibitors, and sensors with advanced (super resolution) imaging modalities, such as PharmacoSTORM and correlative light-electron microscopy, will uncover the fundamental basis of lipid signaling at nanoscale resolution in the brain. Furthermore, chemical biology approaches enable the translation of these fundamental discoveries into clinical solutions for brain diseases with aberrant lipid signaling.

## Key References

- Ogasawara, D.; Deng, H.; Viader, A.; Baggelaar, M. P.; Breman, A.; den Dulk, H.; van den Nieuwendijk, A. M. C. H.; Soethoudt, M.; van der Wel, T.; Zhou, J.; Overkleeft, H. S.; Sanchez-Alavez, M.; Mori, S.; Nguyen, W.; Conti, B.; Liu, X.; Chen, Y.; Liu, Q.-s.; Cravatt, B. F.; van der Stelt, M. Rapid and profound rewiring of brain lipid signaling networks by acute diacylglycerol lipase inhibition.Proc. Natl. Acad. Sci.2016, 113, 26–332666835810.1073/pnas.1522364112PMC4711871.^1^ This paper describes the discovery of DO34 and DH376, the first in vivo active inhibitors of diacylglycerol lipases (DAGL), which reduce the endocannabinoid 2-AG levels and neuroinflammation in the brain.
- van Esbroeck, A. C. M.; Janssen, A. P. A.; Cognetta, A. B., III; Ogasawara, D.; Shpak, G.; van der Kroeg, M.; Kantae, V.; Baggelaar, M. P.; de Vrij, F. M. S.; Deng, H.; Allarà, M.; Fezza, F.; Lin, Z.; van der Wel, T.; Soethoudt, M.; Mock, E. D.; den Dulk, H.; Baak, I. L.; Florea, B. I.; Hendriks, G.; De Petrocellis, L.; Overkleeft, H. S.; Hankemeier, T.; De Zeeuw, C. I.; Di Marzo, V.; Maccarrone, M.; Cravatt, B. F.; Kushner, S. A.; van der Stelt, M. Activity-based protein profiling reveals off-target proteins of the FAAH inhibitor BIA 10-2474. Science2017, 356, 1084–10872859636610.1126/science.aaf7497PMC5641481.^2^ This paper describes the off-target profiling of BIA10-2474, an experimental drug that led to the death of a healthy volunteer in a phase 1 trial, thereby showcasing the importance of activity-based proteomics in the therapeutic development of drugs targeting lipid metabolism.
- Mock, E. D.; Mustafa, M.; Gunduz-Cinar, O.; Cinar, R.; Petrie, G. N.; Kantae, V.; Di, X.; Ogasawara, D.; Varga, Z. V.; Paloczi, J.; Miliano, C.; Donvito, G.; van Esbroeck, A. C. M.; van der Gracht, A. M. F.; Kotsogianni, I.; Park, J. K.; Martella, A.; van der Wel, T.; Soethoudt, M.; Jiang, M.; Wendel, T. J.; Janssen, A. P. A.; Bakker, A. T.; Donovan, C. M.; Castillo, L. I.; Florea, B. I.; Wat, J.; van den Hurk, H.; Wittwer, M.; Grether, U.; Holmes, A.; van Boeckel, C. A. A.; Hankemeier, T.; Cravatt, B. F.; Buczynski, M. W.; Hill, M. N.; Pacher, P.; Lichtman, A. H.; van der Stelt, M. Discovery of a NAPE-PLD inhibitor that modulates emotional behavior in mice.Nat. Chem. Biol.2020, 16, 667–6753239390110.1038/s41589-020-0528-7PMC7468568.^3^ This paper describes the discovery of LEI-401, the first in vivo active inhibitor of NAPE-PLD, which reduces the endocannabinoid anandamide levels in the brain. This inhibitor enabled us to show that NAPE-PLD regulates an endogenous anandamide signaling tone controlling emotional behavior, such as stress and fear extinction.
- Bertheussen, K.; van de Plassche, M.; Bakkum, T.; Gagestein, B.; Ttofi, I.; Sarris, A. J. C.; Overkleeft, H. S.; van der Stelt, M.; van Kasteren, S. I. Live-cell imaging of sterculic acid–a naturally occurring 1,2-cyclopropene fatty acid–by bioorthogonal reaction with turn-on tetrazine-fluorophore conjugates.Angew. Chem., Int. Ed.2022, 61, e20220764010.1002/anie.202207640PMC954630635838324.^4^ This paper describes live cell imaging of an oleic acid analogue containing a 1,2-cyclopropene as a bio-orthogonal click handle using various quenched tetrazine-fluorophores, thereby showing its subcellular distribution. The 1,2-cyclopropene holds great promise for incorporation in various fatty acids, allowing live-cell imaging of lipid distribution.

## 1 Introduction

Historically, lipids were viewed as metabolic and structural membrane components to support neuronal function in the brain. In recent years, a diverse set of signaling lipids has been discovered to interact with specific receptors to regulate many neurophysiological processes. These lipid messengers, such as endocannabinoids, prostaglandins, sphingosine-1-phosphate, and lysophosphatic acid, have emerged as key regulators of neurodevelopment, synaptic plasticity, and inflammation. These lipid messengers fundamentally differ from classical neurotransmitters, most notably, in the way they are synthesized and released. Classical neurotransmission is governed by the release of hydrophilic neurotransmitters, such as glutamate and GABA, from presynaptic vesicles into the synaptic cleft and reuptake by dedicated transporter proteins. In contrast, it is proposed that lipid messengers are not stored in vesicles, but are synthesized on demand, and that their lifespan is regulated by dedicated metabolic enzymes. This implies that the biosynthetic and metabolic rates of the involved enzymes are crucial in determining the flux of lipid messengers, thereby controlling the magnitude and duration of their signaling and physiological response. Consequently, lipid biosynthetic and metabolic pathways are often tightly regulated through post-translational modifications (PTMs), ion-cofactors, protein–protein interactions, and the subcellular localization of their substrates and proteins. Experimental methods used to study classical neurotransmitters, like immunohistochemistry or RNA-expression analyses, are less suitable for lipid messengers, because they fail to capture the spatiotemporal dynamics of enzyme activities regulating signaling lipids. Instead, chemical biological approaches have emerged as the main driving force to study lipid messengers in the brain. The development of small-molecule inhibitors, activity-based probes, and bio-orthogonal lipids has allowed researchers to investigate lipid messengers and their enzymes with a spatiotemporal resolution, which was not previously possible. In this Account, we review how chemical probes can be utilized for investigating lipid signaling systems in the brain. We focus on the development and use of chemical probes to study endocannabinoid signaling by (i) inhibiting the biosynthetic and metabolic enzymes; (ii) visualizing the activity of these enzymes; and (iii) controlling the release and transport of the endocannabinoids.

## 2 Visualizing and Controlling Enzyme Activity

### 2.1 The Enzymatic Machinery Controlling Endocannabinoid Signaling

**Figure 1:**
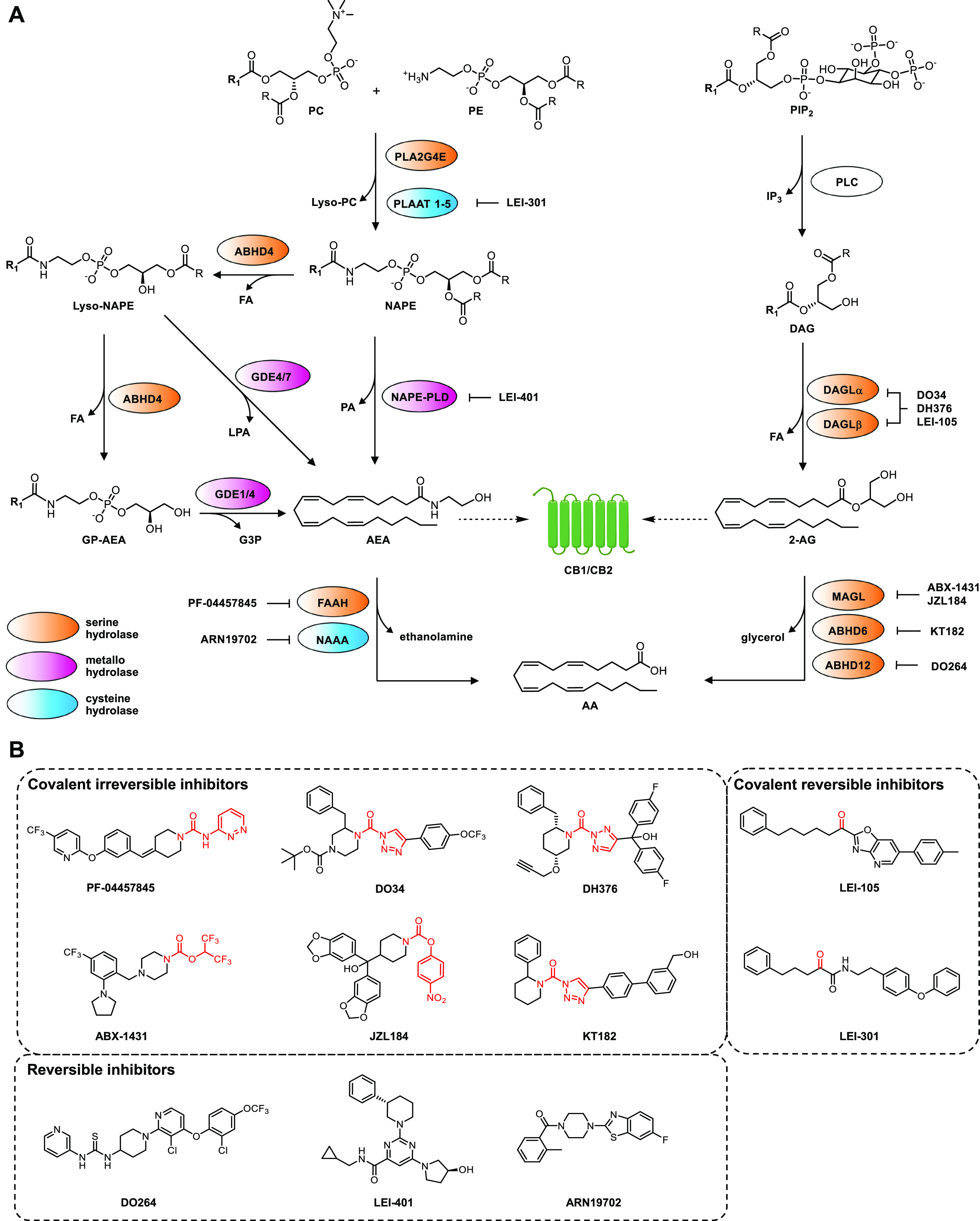
Overview of the metabolic pathways of the endocannabinoid system. (A) R_1_ = C_19_H_31_. Abbreviations: 2-AG, 2-arachidonoylglycerol; AA, arachidonic acid; ABHD, α,β-hydrolase domain containing protein; AEA, *N*-arachidonoyl ethanolamine; CB1/CB2, cannabinoid CB1 or CB2 receptor; DAG, diacylglycerol; DAGL, diacylglycerol lipase; FA, fatty acid; FAAH, fatty acid amide hydrolase; G3P, glycerol-3-phosphate; GDE, glycerophosphodiesterase; GP-AEA, glycerophospho-AEA; IP_3_, inositol 1,4,5-triphosphate; MAGL, monoacylglycerol lipase; NAAA, *N*-acylethanolamine acid amidase; NAPE-PLD, *N*-acylphosphatidylethanolamine phospholipase D; NAPE, *N*-arachidonoylphosphatidylethanolamine; PA, phosphatidic acid; PC, phosphatidylcholine; PE, phosphatidylethanolamine; PIP_2_, phosphatidylinositol 4,5-biphosphate; PLA, phospholipase A; PLAAT, phospholipase A1/2 acyl transferase; PLC, phospholipase C. (B) Chemical structures of the inhibitors depicted in (A). The electrophilic moiety for covalent interaction is indicated in red.

There are two main endocannabinoids: 2-arachidonoylglycerol (2-AG) and *N*-arachidonoylethanolamine (AEA, or anandamide).^5−7^ Both endocannabinoids bind to the cannabinoid CB1 and CB2 receptors to regulate neurotransmission and various forms of synaptic plasticity, such as depolarization-induced suppression of inhibition (DSI) and long-term depression.^8^ Each endocannabinoid has its own independent biosynthetic and metabolic pathway (Figure 1A). 2-AG is synthesized from phospholipids in a Ca^2+^-dependent manner that involves the subsequent actions of phospholipase C and diacylglycerol lipase α or β (DAGLα and DAGLβ).^9^ The majority of the 2-AG pool is inactivated through hydrolysis of the ester bond by monoacylglycerol lipase (MAGL), and to a lesser extent by ABHD6 and ABHD12.^10^

The biosynthesis of AEA is more complex, because it can be synthesized via multiple pathways.^11^ Phospholipase A1/2 Acyl Transferase (PLAAT) 1–5 and PLA2G4E perform the first step in which arachidonic acid from phosphatidylcholine is transferred to the free amine of phosphatidylethanolamine, thereby generating the central precursor *N*-arachidonoylphosphatidyl-ethanolamine (NAPE).^12^ PLA2G4E is the rate-limiting enzyme in neurons that produces NAPEs on demand in a Ca^2+^-dependent manner, whereas the PLAAT enzymes are Ca^2+^-independent and responsible for basal NAPE levels. In the second step, NAPE is hydrolyzed by NAPE-phospholipase D (NAPE-PLD) or, alternatively, by ABHD4 and subsequently GDE1, GDE4, or GDE7, to release AEA.^11^ Fatty acid amide hydrolase (FAAH) is the primary enzyme responsible for the hydrolysis of the amide bond in AEA, thereby inactivating this lipid messenger.^13^*N*-Acylethanolamine acid amidase (NAAA) is also able to hydrolyze AEA, but to a lesser extent.^14^ A complete overview of the enzymatic pathways of the endocannabinoids and their function in the brain has been described in detail in other reviews,^11,15,16^ but there remain many open questions. For instance, is there any cell type or regional specificity of their contributions? Is there a bias for one of the two endocannabinoids to be mobilized: when, where, and why? How does aberrant endocannabinoid signaling lead to disease, and can the inhibitors of endocannabinoid biosynthesis and metabolism be used for therapeutic purposes? The development of chemical probes has contributed to answer some of these questions as outlined below.

### 2.2 Development of Activity-Based Probes to Discover and Map Endocannabinoid Hydrolase Activity

**Figure 2:**
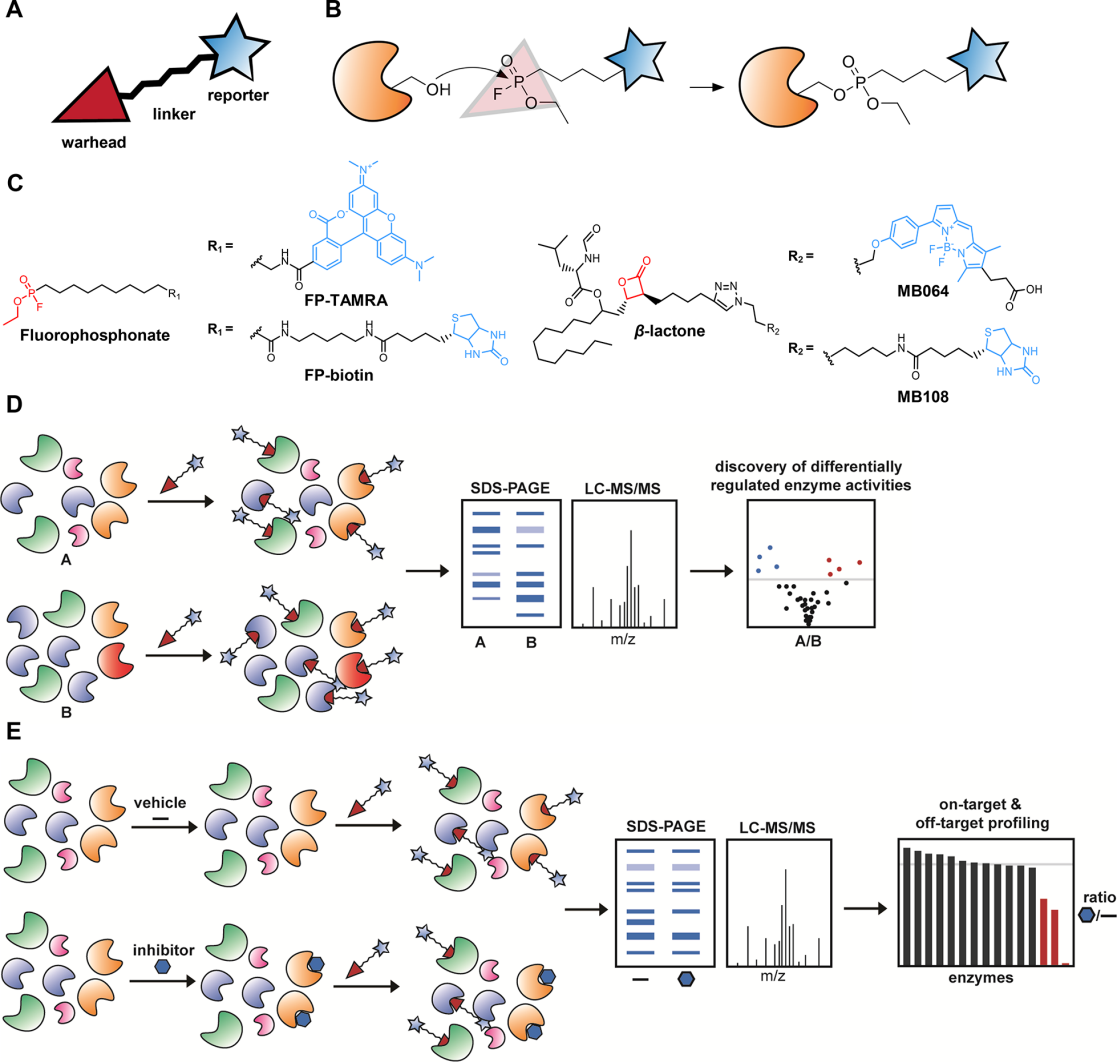
Competitive and comparative ABPP. (A) General schematic overview of a typical activity-based probe. (B) Mechanism of serine hydrolase targeting by fluorophosphonate probes. (C) Structures of fluorophosphonate- and β-lactone probes, equipped with either a fluorophore or a biotin group. (D) General schematic workflow for comparative ABPP. (A) and (B) represent any two biologically different samples. (E) General schematic workflow for competitive ABPP.

The enzymatic machinery of both endocannabinoids predominantly consists of serine hydrolases and cysteine hydrolases (Figure 1A), which use a catalytic serine or cysteine residue, respectively, to hydrolyze esters or amides.^17^ These hydrolases can be targeted by broad-spectrum activity-based probes (ABPs), which were first developed by Cravatt and colleagues in 1999.^18^ An ABP consists of a warhead that covalently reacts with the catalytically active residue of an enzyme in a mechanism-based manner, a linker, and a reporter moiety (Figure 2A). When coupled to fluorescent reporter groups, ABPs enable visualization of enzyme activities in complex proteomes by SDS-polyacrylamide gel electrophoresis (SDS-PAGE). Instead, a biotin reporter group enables affinity enrichment and identification of enzyme activities by mass spectrometry (MS)-based proteomics (chemical proteomics). An ABP informs on the abundance of active enzymes in complex proteomes. The prototypic ABPs for serine hydrolases utilize a fluorophosphonate (FP) as a warhead, which is a broad-spectrum inhibitor of this enzyme family (Figure 2B,C). Recently, we have introduced ABPs with a β-lactone or β-lactam group as alternative warheads to react with hydrolases not targeted by FP-probes, such as DAGLα and cysteine hydrolases (e.g., NAAA and PLAATs).^19−21^ These ABPs can be used in activity-based protein profiling (ABPP) and chemical proteomic experiments in a comparative or competitive setting to assess the functional state of entire enzyme classes in native biological systems.

In comparative ABPP, different biological samples (e.g., healthy vs diseased or different brain regions) are compared to each other to facilitate target identification (Figure 2D). For example, PLA2G4E, ABHD4, ABHD6, and ABHD12 have been discovered using comparative ABPP as enzymes involved in the biosynthesis and metabolism of endocannabinoids.^10,12^ Furthermore, comparative ABPP has been applied to map endocannabinoid hydrolase activity across different brain regions.^22^ This revealed that FAAH activity was highest in the hippocampus, and MAGL activity was most pronounced in the frontal cortex, whereas DAGLα was most active in the cerebellum.^22^ Cell type-specific activities of endocannabinoid hydrolases were studied by Viader et al.^23^ They found a functional segregation of the enzymes across different CNS cell types. For example, high levels of active DAGLα and MAGL were found in neurons, whereas DAGLβ and ABHD12 were mainly found in microglia, suggesting the presence of distinct pools of 2-AG, possibly exerting distinct physiological effects. Comparison of the activity profiles with global expression data revealed a poor correlation, which could indicate post-translational regulation of the endocannabinoid hydrolases. Indeed, FP-based probes are able to capture the Ca^2+^-dependency of PLA2G4E,^12^ showcasing that ABPP can detect dynamic enzymatic activity in relation to the physiological environment.

### 2.3 ABPP Aids the Discovery of Inhibitors to Study Endocannabinoid Function in the Brain

The endocannabinoids are involved in many (patho)physiological functions, such as sleep, appetite, memory formation, anxiety, and pain sensation.^15,16,24^ These biological roles can in principle be studied by genetically modified mice lacking specific endocannabinoid metabolizing enzymes. However, long-term and constitutive inactivation of these enzymes renders the mouse models poorly suited to study rapid and dynamic changes in endocannabinoid signaling. For example, chronic disruption of MAGL results in down-regulation of the CB1 receptor, thereby altering neurotransmission and development of physical dependence.^25,26^ Furthermore, compensatory mechanisms in NAPE-PLD knockout animals have been detected.^27^ Small molecule inhibitors provide, however, a powerful way to assess the temporal consequences of acute enzyme blockade on the physiological response. Because many lipid metabolizing enzymes belong to the class of serine hydrolases, it is essential to have highly selective inhibitors to attribute specific functional roles of lipid metabolizing enzymes.

Competitive ABPP is a powerful technique to characterize inhibitors of lipid metabolizing enzymes (Figure 2E). The inhibitor abrogates labeling of enzymes by the ABP after either in vitro incubation or in vivo treatment. Competitive ABPP enables the possibility to map the on- and off-target activity of small molecules, leads, or drug candidates (and their metabolites) in their native physiological context. To guide inhibitor characterization, we have reported both gel-based and chemical proteomics-based protocols for the evaluation of serine hydrolase inhibitors using ABPP.^28,29^

Competitive ABPP has been instrumental in the discovery of highly selective inhibitors of the biosynthetic and metabolic enzymes of 2-AG and AEA. We have used, for instance, the β-lactone probe MB064 to guide the discovery of the first selective DAGL inhibitor LEI-105, which enabled demonstrating that DSI was dependent on the on demand production of 2-AG in hippocampal slices.^30^ Furthermore, MB064 and FP-probes were employed to guide the discovery of DH376 and DO34, the first centrally active DAGL inhibitors.^1^ These compounds are now widely used to study the role of DAGL in various (patho)physiological processes, such as synaptic plasticity, food intake, neuroinflammation, alcohol and cocaine addiction, epilepsy, stress, and anxiety.^24^ Interestingly, selective DAGLα or DAGLβ inhibitors have not been discovered yet, but would be valuable to understand the contribution of each protein subtype to endocannabinoid signaling in the brain.^31^

Centrally active MAGL inhibitors, such as JZL184 and ABX-1431 (Lu AG06466), were identified and optimized by a competitive ABPP-screen from a library of activated serine hydrolase-directed carbamates.^32,33^ These inhibitors showed efficacy in animal models of inflammatory and neuropathic pain and in various models of neuroinflammatory diseases, including Alzheimer’s disease, Parkinson’s disease, and Multiple Sclerosis.^16^ PF-04457845, a highly selective FAAH inhibitor, was also discovered by ABPP.^34,35^ This compound showed cannabinoid receptor-dependent antinociceptive effects in animal models of inflammatory pain. Although inhibition of FAAH and MAGL in the mouse brain raises the levels of AEA or 2-AG, respectively, neither induces the full spectrum of behavioral changes typically observed by CB1 receptor agonists, such as Δ^9^-tetrahydrocannabinol (THC), the psychoactive constituent of *cannabis sativa*. Interestingly, simultaneous inhibition of both FAAH and MAGL does mimic the behavioral effects of THC. This indicates that 2-AG and AEA have both distinct and overlapping roles in controlling CB1 receptor signaling.^36^ Thus, selective FAAH or MAGL inhibitors may have a therapeutic benefit without inducing the full spectrum of psychoactive effects observed with THC. Currently, ABX-1431 and PF-04457845 are investigated in phase 2 clinical trials for multiple indications, thereby demonstrating the translational value of using ABPP in the drug discovery process.^15^

Another example that showcased the general utility of ABPP in drug discovery was a study in which we reported the selectivity profile of the FAAH inhibitor BIA10-2474.^2^ In a phase 1 clinical trial of the experimental drug, one of the healthy volunteers died, and four others suffered brain damage.^37^ We used competitive ABPP with BIA10-2474 to study its serine hydrolase interaction landscape in human cortical neurons and human brain tissue from subjects not associated with the trial. We found that BIA10-2474 was not selective, but inhibited several different lipases and disrupted cellular lipid networks in cultured neurons.^2^ This emphasizes the need for preclinical selectivity testing in human cells and tissues in an early drug discovery stage and highlights ABPP as a valuable technology to guide therapeutic development.

Finally, competitive ABPP using photoreactive probes enables the profiling of compounds that do not target serine or cysteine hydrolases. These probes lack an electrophilic warhead, but are instead equipped with a photoactivatable cross-linker, which covalently binds the target upon UV irradiation. We have employed such probes to study selective CB2 receptor agonists (vide infra) and cellular target engagement of NAPE-PLD inhibitors.^3,38^ The latter assisted the characterization of LEI-401, the first in vivo active NAPE-PLD inhibitor. Using LEI-401, we demonstrated that AEA biosynthesis in mouse brain was dependent on NAPE-PLD. LEI-401 activated the hypothalamus–pituitary–adrenal axis and impaired fear extinction, thereby emulating the effect of a CB1 receptor antagonist, which could be reversed by a FAAH inhibitor. Our findings highlight the distinctive role of NAPE-PLD in AEA biosynthesis in the brain and suggest the presence of an endogenous AEA tone controlling emotional behavior.^3^

### 2.4 Tailor-Made ABPs Allow One to Study Lipid Metabolizing Enzymes with High Spatial Resolution

Broad-spectrum ABPs have provided an unprecedented view on the enzymatic activity of entire protein families in cells and tissues. However, workflows based on broad-spectrum probes require sample homogenization for analysis, thereby losing spatial information on the (sub)cellular localization of the lipid metabolizing enzymes. Understanding the role of lipid messengers in brain function requires, however, detailed knowledge of their spatiotemporal activities in a region and cell type-specific manner. Therefore, there is a need for tailor-made, highly selective ABPs to visualize lipid metabolizing enzyme activity with enhanced spatial resolution.

To this end, two types of probes can be used. One-step ABPs contain a fluorophore already attached to the probe, which can be used to directly visualize the target. Alternatively, two-step ABPs harbor a bio-orthogonal ligation handle, to which the fluorophore is attached only after the ABP has covalently bound to its target. The archetypical ligation tag for ABPs is the alkyne, which can be coupled to reporter groups via copper-catalyzed azide–alkyne cycloaddition (CuAAC, also known as click chemistry).^39^ This avoids issues of cell permeability and reduced target affinity caused by a fluorophore. Two-step probes usually have improved pharmacokinetic properties and may penetrate the blood–brain barrier. However, direct visualization of enzyme activity by one-step fluorescent probes avoids the secondary ligation step, simplifies the protocol, and is suitable for live-imaging. Both strategies have been employed to study (sub)cellular localization and distribution of active enzymes with confocal microscopy or flow cytometry.^38,40−43^

Multiple one-step probes for visualizing 2-AG metabolizing enzymes have been reported: DH379 and HT-01 for DAGLα and β,^1,44^ and LEI-463-Cy5 and JW912 for MAGL.^45,46^ DH379 was based on the previously mentioned DAGL inhibitor DH376, but has not been applied to visualize DAGL activity in living cells yet. DH379 was, however, instrumental in discovering the short in vivo half live of DAGLα protein (2–4 h), which was rapidly degraded and replaced by newly synthesized enzyme in mouse brain.^1^ This ongoing production of DAGLα generates a strong, tonic flux of 2-AG in the brain. LEI-463-Cy5, based on the selective MAGL inhibitor JW651,^45^ was recently used to visualize the activity of single MAGL molecules in cells by using a super resolution imaging method termed PharmacoSTORM.^46^ Although one-step probes can be used in vivo in the brain by intraventricular injection,^43^ such methods are technically challenging, and therefore two-step probes may be more suitable for in vivo labeling of active enzymes.

**Figure 3:**
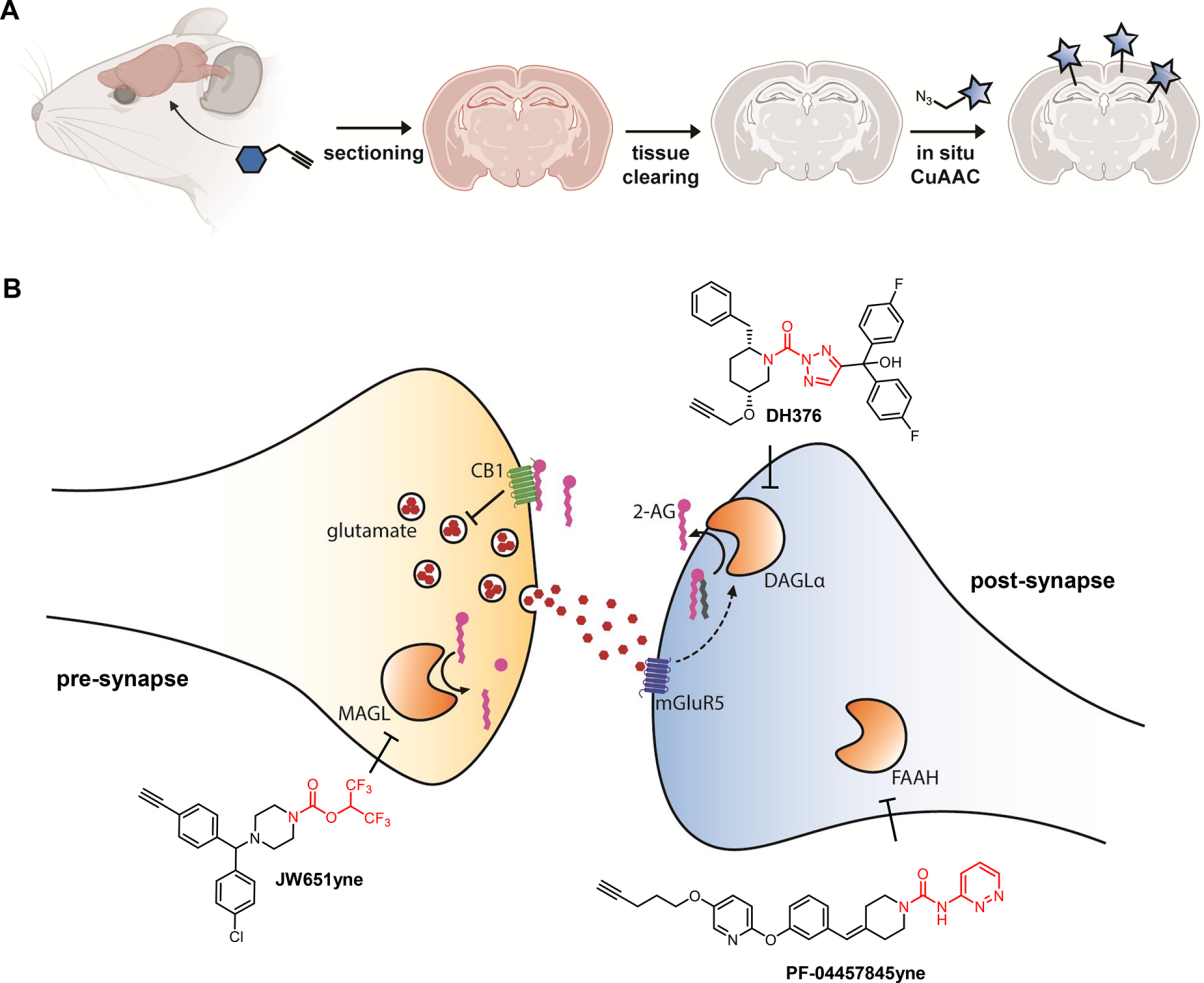
Visualizing nanoscale enzyme activity using activity-based probes. (A) The CATCH workflow for in situ CuAAC. (B) Schematic overview of a synapse with an active endocannabinoid system. Suitable ABPs for targeting pre- and postsynaptic enzymes are shown. (A) was created using biorender.com.

Recently, Pang et al. reported an elegant method to visualize enzyme activity in brain sections using two-step probes. They administered analogues of FAAH inhibitors PF-04457845 and BIA10-2474 equipped with an alkyne to mice, and subsequently coupled the fluorophore ex vivo through CuAAC.^42^ They developed a tissue clearing technique termed CATCH that delipidates the brain using sequential hydrogel-based fixation and detergent-based washing to accelerate the CuAAC reaction (Figure 3A). They showed that FAAH activity was mainly found in the somas of Ctip2+ excitatory neurons in the somatosensory cortex, but not in astrocytes or inhibitory neurons. Furthermore, they visualized the opposing synaptic localization of FAAH and MAGL in post- and presynaptic terminals, respectively, in line with earlier work using immunohistochemistry.^47^ Interestingly, BIA10-2474yne, but not PF-04457845yne, was found to bind to the reticulotegmental nucleus of the pons in FAAH knockout mice, which suggests that off-targets of BIA10-2474 are located in this brain region. The development of novel selective ABPs in combination with the CATCH method offers the exciting opportunity to map active enzymes in the brain with cell type- and synapse-specific resolution, contributing to a spatiotemporal understanding of lipid metabolism at the nanoscale.

## 3 Visualizing and Controlling Lipid Transfer and Action

### 3.1 Lipid Reporter Functionalities

ABPs report on the enzymes that biosynthesize or metabolize lipid messengers, but they do not provide information on the lipid messengers themselves. To this end, it would be desirable to develop tools to directly visualize lipid messengers. Lipids cannot, however, be equipped with genetically encoded fluorescent markers. In addition, due to their small size and hydrophobicity, it has been challenging to directly visualize and quantify the lipid messengers in real-time. Instead, lipids, including 2-AG and AEA, have traditionally been studied through isotopic analogues containing either radioactive (^14^C, ^3^H) or stable (^13^C, ^2^H) isotopes. Yet, these radiolabeled lipid messengers cannot be visualized in a cellular context because they inherently require sample homogenization and are thus mainly restricted to end point measurements. Furthermore, isotopic messengers provide insufficient spatiotemporal resolution to adequately investigate the rapid and local action of lipid signaling.

To circumvent these problems, lipid probes that carry a fluorescent tag have been developed that are suitable for real-time visualization. For example, AEA-TAMRA contains a dye in the ethanolamine headgroup of AEA to visualize cellular uptake.^48^ Alternatively, upon treatment with biotin-AEA and subsequent fixation, streptavidin dyes report on AEA location, and its protein interaction partners were identified through pull down experiments.^49^ However, a major drawback of these tagged probes are the substantial alterations of steric bulk and polarity with respect to the endogenous lipid, and therefore deviating biological effects or artifacts may occur. Thus, these directly tagged lipid messengers should be used with caution to explore poorly characterized aspects of endocannabinoid signaling.

To minimize the negative effect of a tagged analogue, recent research has focused on the introduction of small ligation handles that can be functionalized in downstream analysis through bio-orthogonal chemistry. The previously mentioned alkyne moiety is a popular tag for omega-terminal fatty acid chain functionalization, due to its similarity in size and hydrophobicity with respect to the substituted alkyl group. A variety of alkyne lipids have been employed as probes to study the metabolism of their endogenous counterparts, which are distinguished from the alkyne probe by MS-based methods.^50^ Alternatively, through ligation to a biotin, alkyne lipids that are incorporated into proteins can be enriched to elucidate the lipidation PTM pattern using proteomics.

Alkyne moieties are also incorporated in photoaffinity probes, to identify the biomolecular interaction landscape of signaling lipids. In such probes, following covalent attachment to proximate biomolecules, the alkyne serves as a functionalization handle for enrichment and subsequent proteomics. The Cravatt lab developed two distinct AEA photoaffinity probes to map the AEA interaction protein landscape.^51^ Through this approach, a wide range of novel putative AEA protein interaction partners were identified, including NUCB1, which was found to function in NAE metabolism. In addition, the platform lends itself to in situ pharmacological characterization of the discovered AEA targets. Recently, we exploited this design to investigate the AEA reuptake inhibitor WOBE437.^52^ By using a photoaffinity-WOBE437 probe, we identified previously unknown off-targets of WOBE437. Although these proteins preferentially bound AEA, they were not responsible for the cellular uptake of AEA. These results illustrate the importance and utility of photoaffinity lipids to profile inhibitor selectivity.

Importantly, even an omega-terminal alkyne can profoundly influence the biological behavior of the probe in comparison to the endogenous lipid. For instance, an arachidonic acid alkyne probe was processed differently in eicosanoid metabolism and had weaker immuno-stimulatory effects.^53^ These results emphasize that the altered behavior of a tagged substrate and its metabolites within a biological context is not limited to perturbed enzyme recognition, but may include modulatory effects on endogenous receptors. In addition, the visualization of alkyne lipids has largely been restricted to end-point analysis, because their application to living systems is limited due to the requirement of cytotoxic Cu(I) for the CuAAC.^54^

**Figure 4:**
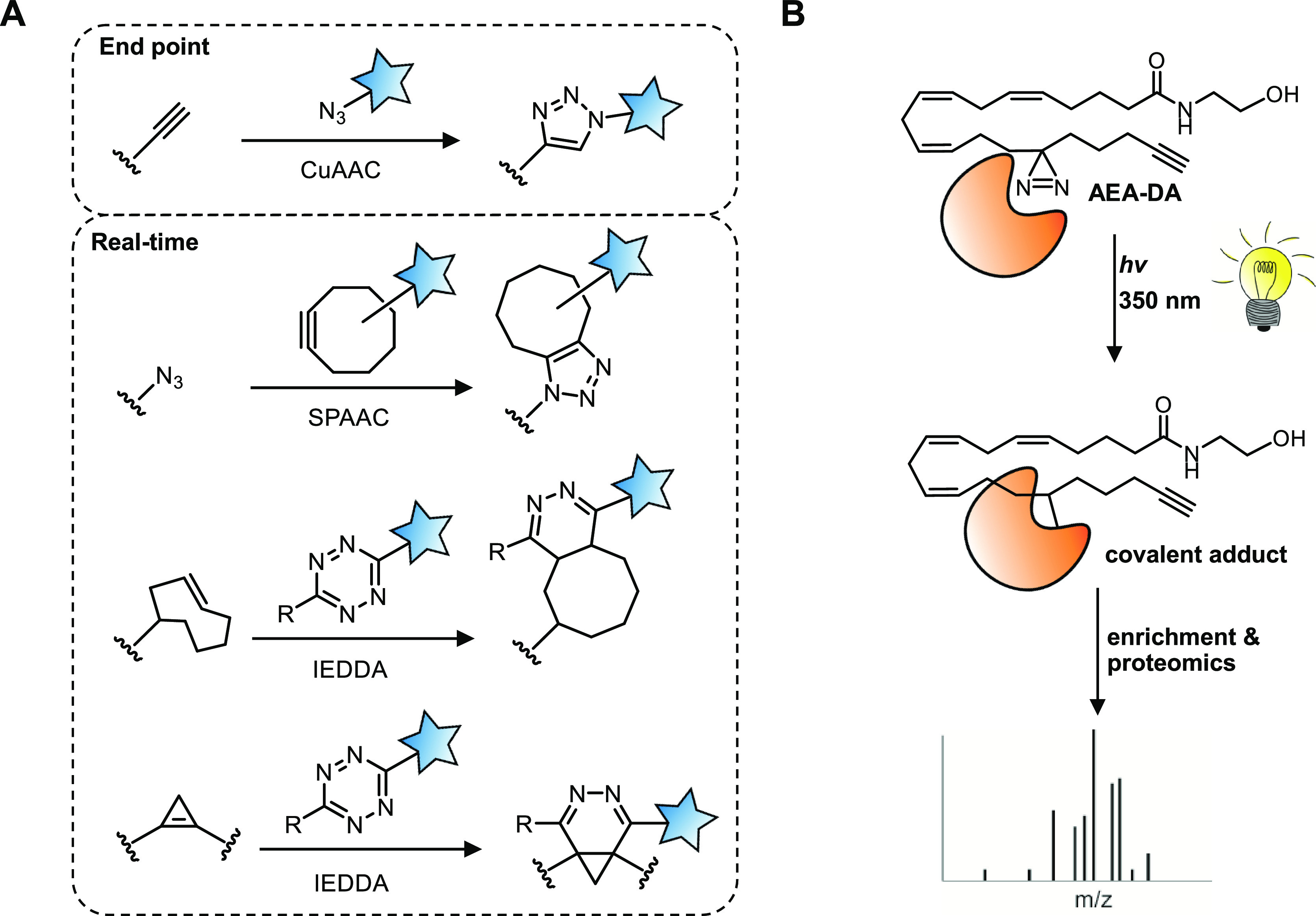
An outline of lipid functionalization strategies. (A) Bio-orthogonal reactions used to functionalize lipids. (B) An AEA photoaffinity probe (AEA-diazirine) to identify the interaction partners of AEA.

Conversely, strain promoted alkyne azide cycloaddition (SPAAC) does not require additional reagents as it relies on the energetically favored ligation of a cyclooctyne to an azide (Figure 4A). Similarly, the inverse electron demand Diels–Alder (IEDDA) occurs between an electron-poor diene, usually a tetrazine, and an electron-rich alkene. The IEDDA has superior ligation kinetics and thus improved temporal resolution over SPAAC. Although both SPAAC and IEDDA may rely on relatively bulky tags, these can be supplied as exogenously labeled precursors, which are then metabolically incorporated into the lipid of interest. Taumara and colleagues utilized azido-choline and spatially restricted cyclooctyne fluorophores to detect organelle-specific phosphatidylcholine lipid transport in live cells.^55^ Likewise, the group of Baskin has exploited the promiscuity of PLD enzymes to transphosphatidylate bio-orthogonal alcohols for the real-time visualization of PLD activity and their phospholipid substrates.^56^

Recently, we expanded the IEDDA substrate scope using sterculic acid, a natural oleic acid analogue containing a 1,2-cyclopropene as a bio-orthogonal click handle, for live cell imaging employing various quenched tetrazine-fluorophores.^4^ This allowed us to visualize its distribution in live cells and capture sterculic acid-modified proteins.

### 3.2 Control over Spatiotemporal Release of Lipid Derived Tools

In addition to visualizing lipid species, there is a need to control the spatiotemporal release of lipids. This is especially relevant for the endocannabinoids, because these lipids will immediately elicit a response when introduced to a biological context, making it challenging to record their initial effect.

**Figure 5:**
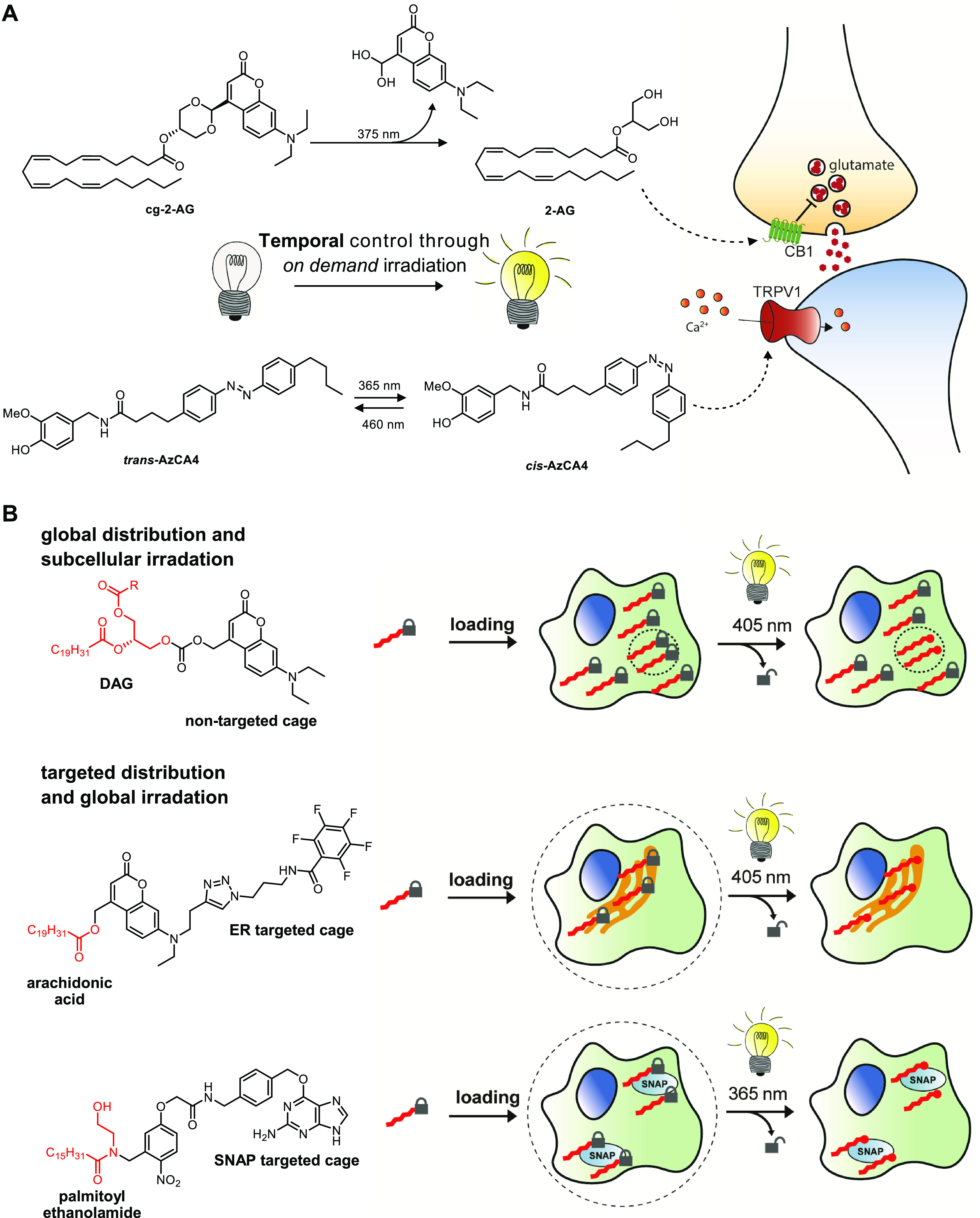
Controlling the spatiotemporal aspects of the lipid probes. (A) Lipids can be functionalized with either a photocleavable cage (cg-2-AG) or a photoswitchable azobenzene (*cis*-AzCA4). Upon irradiation, the probe is converted to its active form to exert its biological function. (B) Spatial control over lipid release can be achieved through caged-lipid strategies with either targeted irradiation or a subcellularly targeted cage.

A prominent design to release lipids on demand is to attach a photoliable protecting group that renders the initial molecule biologically inactive (Figure 5A). Upon irritation at the appropriate wavelength, this photocage is removed, and the initial response to the bioactive molecule can be recorded. As early as 2005, Heinbockel and colleagues synthesized an AEA analogue with a nitrobenzoyl protecting group.^57^ Using this tool, they were among the first to recognize the millisecond time scale of endocannabinoid signaling. Recently, the Schultz lab has expanded this principle to a coumarin protected 2-AG,^58^ nitrobenzoyl protected DAGs,^59^ and a coumarin caged DAG photoaffinity probe.^60^ Notably, the latter trifunctional probe identified many DAG interaction partners that were not previously captured through standard chemical proteomics strategies. This suggests that photocaged probes allow one to study more short-lived and low affinity interactions.

Alternatively, photoswitchable probes may also be used to study the rapid cellular responses of lipid messengers (Figure 5A). Typically, an azobenzene moiety will be incorporated in the hydrophobic tail of the lipid, which undergoes *trans* to *cis* isomerization upon light irradiation. The *cis*-azobenzene mimics the bend conformation of poly unsaturated fatty acids, while *trans*-azobenzene more closely resembles saturated fatty acids. This has motivated the development of probes to control the activation of the ion channel TRPV1^61^ and protein kinase C.^62^ Recently, Frank and co-workers have created photoswitchable probes for the on demand activation of the CB1^63^ and CB2 receptors.^64^

Photocaged and -switchable lipid probes also provide spatial control through irradiation of particular cellular areas in the biological specimen (Figure 5B). Wagner et al. have extended this concept by designing coumarin-caged arachidonic acid probes containing an additional moiety that targeted the probe to specific organelles.^65^ Precise targeting was also achieved with an optically cleavable targeted palmitoylethanolamide (OCT-PEA) analogue by Tobias et al.^66^ OCT-PEA carried a guanine motif that binds to a genetically encoded spatially restricted SNAP-tag and thus in theory could be targeted to any membrane or protein. OCT-PEA was, however, only located at the plasma membrane, due to its membrane impermeability, where it was released to function as a GPR55 agonist.

In conclusion, the development of photocaged and -switchable probes has proven useful to control the release and function of the signaling lipid on demand, thereby allowing one to study the acute cellular responses of the probe.

### 3.3 Spatiotemporal Readout of Lipid Action

**Figure 6:**
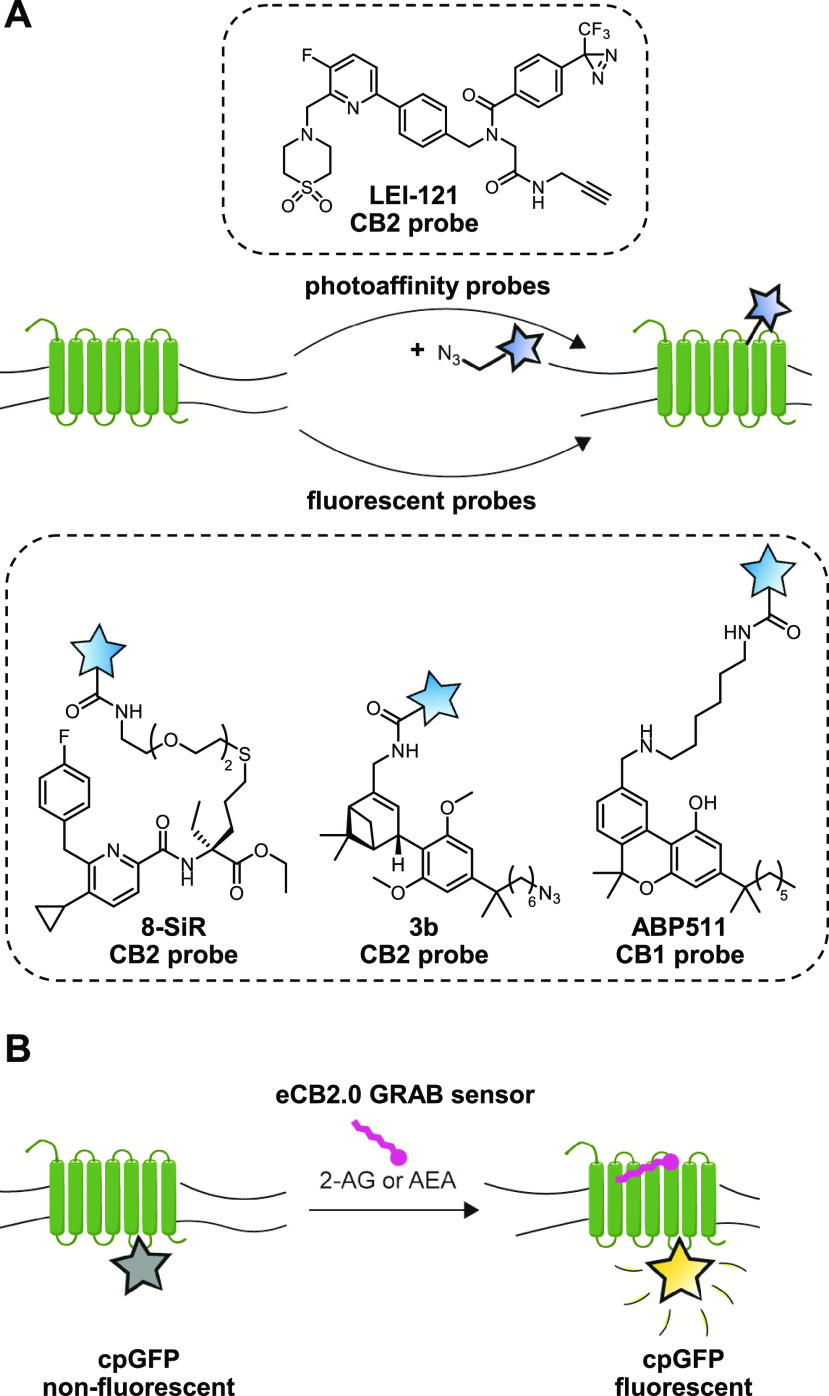
Visualizing lipid receptor localization and activation. (A) CB1 and CB2 receptors can be targeted with either a two-step photoaffinity probe or a directly fluorescent probe to reveal its location and occupancy. (B) The genetically encoded eCB2.0 receptor contains a circularly permutated GFP (cpGFP), which will only become fluorescent upon binding 2-AG or AEA to report on endocannabinoid action.

Following the precisely controlled release of lipids, it is paramount to have tools with equivalent spatiotemporal capability to record their action on biological systems. Although there are well-established tools available for electrical recordings and the characterization of second messengers, it has been more challenging to directly visualize CB1 and CB2 receptor activation or location (Figure 6). Our lab developed LEI-121, a CB2 receptor selective probe that harbors a diazirine for covalent cross-linking and an alkyne for downstream functionalization.^38^ As the first GPCR photoaffinity probe, LEI-121 lends itself useful for pharmacological profiling of CB2 ligands via SDS-PAGE and proteomics. In addition, reversible fluorescent CB2 probes with improved spatiotemporal properties have been developed and applied to in vivo zebrafish models.^41,67^ For the CB1 receptor, a similar probe was developed by Katona and colleagues, which enabled single-molecule receptor visualization in HEK-293 cells using PharmacoSTORM.^46^

Contrary to a ligand-based approach, GPCR activation-based (GRAB) sensors exploit the conformational changes associated with GPCR activation through the introduction of a circularly permutated GFP, which will fluoresce upon ligand binding (Figure 6B). The development of a CB1 receptor-based GRAB sensor (eCB2.0) by Li and colleagues is, arguably, a major breakthrough in the field of endocannabinoid research.^68^ This real-time eCB2.0 reporter provides a readout for 2-AG and AEA signaling at high spatiotemporal resolution in cultured neurons, acute brain slices, and in vivo mice models.^68^ They could show, using multiplexed in vivo Ca^2+^ and eCB2.0 recordings, that spontaneous neural activity and endocannabinoid signaling in the hippocampus are highly synchronous.^69^ Acutely blocking either DAGLα/β or MAGL, but not FAAH or NAPE-PLD, using selective inhibitors, disrupted this synchrony, showing this was mediated via the on demand release of 2-AG, and not AEA.

The combination of genetically encoded sensors such as eCB2.0 and control over lipid activation, metabolism, and localization will allow the deciphering of endocannabinoid signaling with nanoscale spatial and millisecond temporal resolution.

## 4 Summary and Outlook

Lipid signaling has been inherently difficult to study because of the hydrophobicity of lipid messengers, their rapid metabolism, and the promiscuity of the metabolic networks controlling these lipids. In this Account, we have described chemical biology approaches to control and visualize lipid messengers, specifically the endocannabinoids, in the brain.

The extensive use of ABPP in this field has resulted in the development of excellent chemical probes to perturb and visualize enzyme activity in vitro and in vivo. Highly selective inhibitors are required to decipher the functional roles of the various enzymes in lipid networks. Additionally, these will form the basis to develop the next generation in vivo active ABPs. Combined with recent technical advances in visualization, such as CATCH, PharmacoSTORM, and correlative light-electron microscopy, this will allow researchers to visualize enzymatic activity with unprecedented resolution and specificity.

Lipid probes with photocaged or photoswitchable moieties can be released on demand in a spatially controlled manner, thereby allowing one to study the acute cellular responses of signaling lipids. Next generation lipid probes should be developed for live cell compatibility, real-time visualization, and minimal structural deviation from the endogenous lipid. Future exploration of the bio-orthogonal reaction space should be focused on the physiological environment in which lipids reside such as the plasma membrane or the endoplasmic reticulum.

The release of endogenous endocannabinoids can now be monitored in high spatiotemporal resolution with the GRAB sensor eCB2.0. These genetically encoded tools report on real-time endocannabinoid signaling and will complement the ABPs and lipid probes to complete our tool set for imaging lipid signaling. GRAB sensors that are specific to 2-AG or AEA activation are currently under development and would be highly valuable to dissect the individual roles of 2-AG and AEA in the brain.

To conclude, the lack of appropriate tools to study lipid signaling has long hampered their study as compared to other biomolecules. Recent advances in the chemical biology of lipids now provide researchers with the tools to more accurately track lipid metabolism, location, and action. Together with innovations in the fields of bioimaging, these will form the future basis to uncover the underlying mechanisms of lipid signaling. Finally, it is also envisioned that the chemical biology approaches described in this Account will facilitate the translation of the fundamental discoveries into clinical solutions for brain diseases with aberrant lipid signaling.
